# Effects of the Amount and Frequency of Fluid Intake on Cognitive Performance and Mood among Young Adults in Baoding, Hebei, China: A Randomized Controlled Trial

**DOI:** 10.3390/ijerph17238813

**Published:** 2020-11-27

**Authors:** Hairong He, Jianfen Zhang, Na Zhang, Songming Du, Shufang Liu, Guansheng Ma

**Affiliations:** 1 Department of Nutrition and Food Hygiene, School of Public Health, Peking University, Beijing 100191, China; hehairong_16@bjmu.edu.cn (H.H.); zjf@bjmu.edu.cn (J.Z.); zhangna@bjmu.edu.cn (N.Z.); 2 Chinese Nutrition Society, Beijing 100053, China; dusm9709@126.com; 3 Health Science Center, Hebei University, Baoding 071000, China; shufangliu@126.com; 4 Laboratory of Toxicological Research and Risk Assessment for Food Safety, Peking University, Beijing 100191, China

**Keywords:** fluid intake amount, fluid intake frequency, cognitive performance, mood, young adults

## Abstract

Water is a critical nutrient that is important for the maintenance of the physiological function of the human body. This article aimed to investigate the effects of the amount and frequency of fluid intake on cognitive performance and mood. A double-blinded randomized controlled trial was designed and implemented on college students aged 18–23 years in Baoding, China. Participants were randomly assigned into one of three groups: the recommended behavior group (RB group) who drank 200 mL of water every 2 h, the half amount group (HA group) who drank 100 mL of water every 2 h, and the high frequency group (HF group) who drank 110 mL of water every 1 h. The intervention lasted 2 days. Urine osmolality, cognitive performance, and mood of participants in each group were compared using the one-way analysis of variance (ANOVA). A total of 92 participants (46 females, 46 males) completed this study with a completion rate of 95.8%. The urine osmolality of the HA group was higher than that of the RB group and the HF group at two time points (*p* < 0.05). At time point 1, the scores in the portrait memory test and vigor were statistically different (*F* = 20.45, *p <* 0.001; *F* = 5.46, *p =* 0.006). It was found that the scores for the portrait memory test in the RB group were lower than those in the HA group and the HF group (*p =* 0.007; *p <* 0.001), while the scores of the HF group were higher than those of the HA group (*p <* 0.001). The scores for vigor in the RB group were significantly higher than those of the HA group (*p =* 0.006), and they were also significantly higher than those of the HF group (*p =* 0.004). At time point 2, only the scores for vigor were statistically different (*F* = 3.80, *p =* 0.026). It was found that the scores for vigor in the RB group were higher than those in the HA group and HF group (*p =* 0.018; *p =* 0.019). Both the amount and frequency of fluid intake may affect urine osmolality and vigor, but these factors have limited impacts on cognitive performance. Rational fluid intake behavior may be beneficial to improve the hydration status and mood of young adults. More research is needed, especially experimental research, to allow causal conclusions to be drawn.

## 1. Introduction

Water is a vital nutrient that is required for humans to survive, accounting for 60–70% of an adult’s bodyweight [1]. In the human body, water has many physiological functions and plays important roles in health promotion and disease prevention. Insufficient or excessive water intake can have negative impacts on health [2,3,4,5,6,7,8,9]. Sources of water in the body include drinking fluids, water from food, and endogenous water, among which fluid intake is the most important source [10].

The total amount of water in human body is maintained in a dynamic balance, i.e., the intake of water is about the same as the amount of water discharge [11]. When people discharge far more water than they take in, dehydration can occur. Previous studies about hydration among adults in China showed that only a quarter of people had an optimal hydration status (urine osmolality ≤ 500 mOsm/kg), which means that they were meeting daily fluid intake recommendations and ensuring sufficient excretion of urine to reduce the risk of kidney disease [12,13,14]. As the degree of dehydration increases, the amount of harm to the body increases. Even when fluid loss is less than 1% of body weight, it will have negative effects on brain function and mood [15,16]. When the amount of fluid loss is about 1% of bodyweight, the plasma osmolality increases and there is a feeling of thirst [17]. When water loss accounts for 2% to 4% of bodyweight, the urine color will darken and there will be reduced work efficiency. If the water loss exceeds 8% of bodyweight, mental and nervous system abnormalities will occur, and when it is 20%, death can occur [18].

Recently, growing evidence has shown that dehydration impacts cognitive performance negatively in both children and adults. Through a literature search, Merhej reported that even mild dehydration is associated with impairments in cognitive function [19]. Similarly, Stachenfeld’s findings demonstrated that mild dehydration could lead to impairment in the visual and working memory and executive function in healthy young women, and these effects could be reversed after drinking water [20]. However, studies have obtained inconsistent conclusions. A crossover trial conducted in schools in Mali indicated that drinking water did not improve the cognitive performance of children, and the impact was masked by the practice effect—that is, the impact of training for cognitive tests masked the impact of the intervention [21].

Fluid intake behavior includes the amount and frequency of drinking fluids. Since the scientific evidence is not robust and is not suitable for the general population, only few guidelines mention fluid intake behavior, i.e., drinking water frequently in small quantities [1]. A few studies have been conducted on the frequency of fluid intake. These have mainly focused on the frequency of fluid intake of athletes during or after exercise. The conclusions of these studies on the rate of fluid intake have been inconsistent. Athletes were once be recommended to drink water at a certain rate [22]. However, in recent years, there have been several studies and case reports recommending ad libitum fluid intake but setting an hourly rate [23,24]. Kovacs also observed that after dehydration to 3% of bodyweight following cycling exercise, plasma volume and fluid balance restored faster when athletes drank at a high rate over a period of 3 h than when the same amount of fluid was drunk in 5 h [25]. Although the relevant evidence for the general population is limited, it can be seen that fluid intake frequency may impact fluid balance.

The purpose of this study was to investigate the effects of different amounts and frequencies of fluid intake on cognitive performance and mood among young adults and to provide a scientific basis for the appropriate fluid intake behavior for young adults.

## 2. Materials and Methods

### 2.1. Participants Recruitment

Participants were recruited from Hebei University Health Science Center, Baoding, China. The inclusion criteria were as follows: healthy male and female college students aged between 18 and 23 years old. The exclusion criteria were as follows: participants aged <18 years or >23 years; menstruating female college students; participants who habitually smoked, drunk alcohol (>20 g/day), or consumed large amounts of caffeine (>250 mg/day); and participants who had oral, endocrine, kidney, gastrointestinal, or metabolic diseases [26]. Recruitment notices were sent through WeChat and QQ (Tencent Holdings Ltd., Shenzhen, China) (instant messaging apps widely used by college students). A participant recruitment campus talk that all college students could participate in was also held. All subjects had the right to accept or refuse to participate in research anonymously. Students who were interested in this research voluntarily applied and provided related basic information, such as medical records, so that the investigators could screen them according to the inclusion and exclusion criteria. The selected participants signed the informed consent forms to join the study, and a further medical examination was arranged to exclude participants in a diseased state.

### 2.2. Sample Size Calculation

The following formula was used to calculate the sample size: α was set at 0.05, and the power was set at 0.8. *σ* and *d* were set at 7.3 and 5.5, respectively [27]. Considering the possible dropout rate, the sample size was increased by 10% on the basis of formula calculation, that is, 30 participants were needed in each group—half males and half females.

$$n\;=\;\frac{2{\left(Z_{\alpha }+Z_{\beta }\right)}^{2}\sigma^{2}}{d^{2}}$$

### 2.3. Ethical Review

The study protocol was approved by the Peking University Biomedical Ethics Committee and registered in the Chinese clinical trial registry (ChiCTR-IOR-17011568). The ethical approval project identification code was IRB00001052-16071. The whole study process was conducted in accordance with the ethical standards laid down in the 1964 Declaration of Helsinki and its later amendments. All participants signed informed consent forms prior to their inclusion in the trial.

### 2.4. Study Procedure

**Figure 1:**
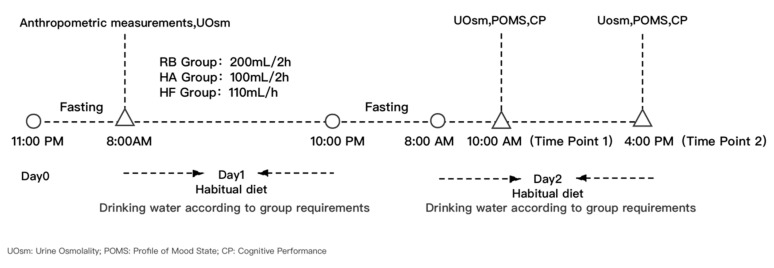
The study procedure.

Participants were randomly assigned into one of three groups using random numbers generated by computer software: participants in the recommended behavior group (RB group) drank 200 mL of water every 2 h, those in the half amount group (HA group) drank 100 mL of water every 2 h, and those in the high frequency group (HF group) drank 110 mL of water every 1 h. The fluid intake behavior of the RB group was formulated according to the fluid intake recommendations in the Dietary Guidelines for Chinese Residents (2016) [28]. Participants were asked not to eat any food or drink any fluid between 11 p.m. on the day before the intervention and 8 a.m. on the first day of the trial. During the trial, participants could eat as usual without intervention. At 8:00 a.m. on the first study day, anthropometric data and urine osmolality were collected. From 8:00 a.m. to 10:00 p.m. on the first study day, participants consumed purified water (provided by the school drinking water system) according to the instructions, and this was repeated from 8:00 a.m. to 4:00 p.m. on the second study day. Thus, participants in the RB group drank 200 mL 8 times a day, i.e., 1600 mL in total for the day. The HA group drank 100 mL 8 times a day, i.e., 800 mL in total for the day. The HF group drank 110 mL 15 times a day, i.e., 1650 mL in total for the day. Drinking water instructions include drinking water according to the requirements of the group, and amount of water in the cup needed to be photographed before and after drinking and sent to the supervisors of each group. The investigators were also responsible for reminding the group members to drink water before the time point of each group. Relevant indicators about cognitive performance and mood, as well as urine osmolality, were collected twice at 10 a.m. (time point 1) and 4 p.m. (time point 2) on the second study day. The study procedure is shown in Figure 1. Computer-based randomization was performed by an unblinded investigator to allocate participants to each group. The data on group assignment were stored separately by the designated investigator who was not involved in participant recruitment, assessment, or analysis. Throughout the course of the trial, the group information was always kept confidential to the participants and data collectors. At the same time, participants were not allowed to talk to each other about their fluid intake patterns. The results of the cognitive performance tests, mood assessment, and urine samples were only labelled with numbers corresponding to participants. If participants dropped out during the trial, their reasons for withdrawal were recorded, but their data were not included in the final analysis.

### 2.5. Anthropometric Measurements

At 8 a.m. on the first day of the trial, height and weight were measured twice to the nearest 0.1 cm and 0.1 kg by trained investigators following standardized procedures with a height–weight meter (HDM-300; Huaju, Zhejiang, China), with the participants wearing light clothes and no footwear. Body mass index (BMI) was calculated using the following equation: [BMI = weight (kg)/height squared (m^2^)].

### 2.6. Assessment of Hydration Status

The urine of participants was collected and tested for osmolality using the freezing point method with an osmotic pressure molar concentration meter (SMC 30C; Tianhe, Tianjin, China). Dehydration is defined as a urine osmolality of greater than 800 mOsm/kg [29]. Optimal hydration is defined as urine osmolality ≤ 500 mOsm/kg [14]. When the urine osmolality is >500 mOsm/kg and ≤800 mOsm/kg, the status is defined as euhydration.

### 2.7. Assessment of Cognitive Performance

Cognitive performance was tested with the “cognitive ability test”, a set of tests developed by the Institute of Psychology, Chinese Academy of Sciences, which consists of 5 modules: the digit symbol substitution test, number cancellation test, working memory span test, portrait memory test, and paper-folding test. To avoid the impact of practice bias, the test was based on validated scales and used the principle of randomization to guarantee that the stimuli were not repeated. The purposes and methods of each test were as follows.

The digit symbol substitution test was used to measure the processing speed of the participants [30]. There were 9 numbers from 1 to 9 with a symbol below each number (“the translation table”). Participants were asked to fill in the corresponding symbol in the space under the randomized number according to the translation table from left to right, and skipping was not allowed. Finally, the correct number of symbols filled in was calculated, with each symbol counting for 1 point. The more correct symbols a participant wrote in 90 s, the higher their score was and the better their processing speed was.

The number cancellation test was printed and was used to assess participants’ ability to simultaneously target stimuli while ignoring distractors. This test was adapted from the K-T cancellation test, which has been frequently used in other studies [31,32]. The participants were asked to cross out all numbers between 3 and 7, or between 7 and 3, from left to right and from top to bottom as rapidly and accurately as possible in 3 min. The participants were asked to draw a terminator on the last number processed when the administrator told them to stop. Scoring consisted of the number of correct cancellations as well as the rate.

The working memory span test included a mental arithmetic section and a vocabulary memory section and was used to evaluate the processing and temporary storage ability [33]. Participants were asked to do a mental arithmetic task and remember the vocabulary shown to them afterwards. During the process of presenting formulas and vocabulary, participants were only allowed to remember words in their minds and were not allowed to write them down or make marks. There were 5 groups of stimuli in total. Each group contained 2 sets of questions, and the number of questions posed to each group increased from 2 to 6. Participants calculated the correct results and were awarded 1 point for repeating the correct vocabulary; higher scores indicated better ability.

The portrait memory test was performed to evaluate the episodic memory ability. This was adapted from the Rivermead Behavioral Memory Test [34]. Six portraits were shown, as well as their surnames, occupations, and hobbies. The participants were asked to recall the description of the portrait. Then, the test process was repeated. When participants answered with the right surnames, occupations, and hobbies of the portrait, they were given scores of 2, 1, and 1, respectively.

The paper-folding test, a dynamic spatial transformation test, was performed to assess the participants’ spatial ability [35]. Participants were asked to imagine that a flat square of paper was folded at least once with a hole punched in it. They needed to mentally unfold the paper and choose the option that matched the positions of holes in the paper. The test had 12 sections that gradually increased in difficulty. Participants were awarded 1 point for each correct answer—the more correct choices, the higher the score, and the better their ability was predicted to be.

### 2.8. Assessment of Mood

The Abbreviated Profile of Mood State (POMS) was used to evaluate mood [36]. This test consists of 40 adjectives that refer to 7 different mood states: tension, anger, fatigue, depression, confusion, vigor, and esteem. Participants were asked to choose the degree of each adjective on a 5-point Likert scale format (“not at all” to “extremely”), in response to the question, “How are you feeling right now?” Through summing the scores for tension, depression, fatigue, confusion, and anger and then subtracting the sum of the scores for vigor and esteem and finally adding a constant of 100, we obtained the total mood disturbance (TMD) score. A higher TMD score reflected a more negative mood state.

### 2.9. Temperature and Humidity of the Environment

The temperature and humidity may also affect participants’ hydration status, cognitive performance, and mood. Therefore, related records were needed to ensure that the temperature and humidity were not extreme or had undergone obvious fluctuation. The temperature and humidity at the study site, both indoors and outdoors, were measured using a temperature hygrometer (WSB-1-H2, Exasace, Zhengzhou, China) during the trial.

### 2.10. Statistical Analysis

SPSS Statistics 24.0 (IBM Corp., Armonk, NY, USA) was used for the statistical analysis. Quantitative variables were tested for normality using normal quantile plots and the Shapiro–Wilk tests of normality. The quantitative parameters of the participants were presented as the mean ± standard deviation (SD) for data that were normally distributed. One-way analysis of variance (ANOVA) combined with the least significant difference method (LSD) were used to explore the differences in normally distributed data among three groups. The distribution of hydration status was expressed in frequency and percentage. Chi-square tests were used to compare the differences in hydration status. A two-sample *t*-test was used to analyze the differences in temperature and humidity during the test. Two-sided significance levels were set at 0.05 (*p <* 0.05) with 95% confidence intervals (95% CI).

## 3. Results

### 3.1. Characteristics of the Participants

A total of 96 participants were recruited, and 92 (46 males and 46 females) completed the study, with a completion rate of 95.8%. The remaining four participants voluntarily withdrew from the trial for personal reasons. The proportions of male participants in the RB group, HA group, and HF group were 52%, 50%, and 48%, respectively. At baseline, no statistically significant differences in age, height, weight, and BMI were found among groups. The characteristics of the participants are shown in Table 1.

### 3.2. Temperature and Humidity

The average indoor and outdoor temperatures during the trials were 25.0 and 25.1 °C, respectively, while the average indoor and outdoor humidity were 72% RH and 76% RH, respectively. Specific values are shown in Table A1 in Appendix A. Since there were no statistical differences in the environmental temperature and humidity within the two days (*p* > 0.05), these parameters were not included in the following analysis.

### 3.3. Effects of Different Amounts and Frequencies of Fluid Intake on Hydration Status

It was found that the proportions of dehydrated people in each group all exceeded 20% (20.7%, 28.1%, and 29.0%) at baseline. No significant differences in urine osmolality or the distribution of hydration status were found among three groups (*F* = 0.22, *p =* 0.800; $\chi^{2}\;$ = 2.96, *p =* 0.564) at baseline. The differences in urine osmolality of participants among the three groups were statistically different at the two time points (*F* = 16.56, *p <* 0.001; *F* = 9.16, *p <* 0.001), and the LSD test was performed. The results showed that the urine osmolality of the HA group was higher than that of the RB group at the two time points, and the difference was statistically significant (*p <* 0.001; *p =* 0.004). The urine osmolality of the HA group was also higher than that of the HF group (*p <* 0.001; *p <* 0.001). There was a statistical difference in the hydration status distribution between the RB group and HA group at time point 1 ($\chi^{2}\;$ = 14.70, *p =* 0.006). The distribution of hydration status in the HA group was more unfavorable for maintaining health. No statistical differences were found between the other groups at time points 1 and 2. The urine osmolality and hydration status distribution of the three groups are shown in Table 2.

### 3.4. Effects of Different Amounts and Frequencies of Fluid Intake on Cognitive Performance and Mood

At time point 1, the comparison of the three groups showed statistically different scores for the portrait memory test and vigor (*F* = 20.45, *p <* 0.001; *F* = 5.46, *p =* 0.006). After the post hoc analysis, it was found that the scores for the portrait memory test were lower in the RB group than in the HA group and the HF group (*p =* 0.007; *p <* 0.001), while the scores in the HF group were higher than those in the HA group (*p <* 0.001). The differences were statistically significant. The vigor scores in the RB group were significantly higher than those in the HA group (*p =* 0.006) and significantly higher than those in the HF group (*p =* 0.004).

At time point 2, the comparison of the three groups showed that only the vigor scores were statistically different (*F* = 3.80, *p =* 0.026). After post hoc analysis, it was found that the vigor scores in the RB group were higher than in the HA group and the HF group (*p =* 0.018; *p =* 0.019), while the difference between the HA group and HF group was not statistically different. The cognitive performance and mood scores of the participants from the three groups are shown in Table 3 and Table 4.

## 4. Discussion

There is a consensus among researchers that reasonable fluid intake behavior is of great significance for the maintenance of human health. However, the specific aspects of the impact of fluid intake behavior have not been confirmed. This study explored the effects of different amounts and frequencies of fluid intake on hydration status, cognitive performance, and mood of young adults, and our findings indicate that fluid intake amount and frequency could affect hydration status and mood.

The results are consistent at two time points, that is, the urine osmolality of the RB group and the HF group were found to be lower than that of the HA group, which shows that the urine osmolality is mainly affected by the amount of fluid taken in, rather than the frequency of fluid intake. The result is similar to the findings of a previous survey performed in college students in Hebei, which also verified that insufficient fluid intake leads to an increase in urine osmolality [27]. The mechanism by which a decrease in fluid intake amount leads to an increase in urine osmolality may be the increased reabsorption of water by the kidneys, resulting in decreased urination. Since the total number of solute particles remains unchanged, the urine osmotic pressure will increase.

Vigor is a positive mood state in the POMS test, which can be described as cheerful, active, and energetic—the higher the score, the more positive the participants are. The results for this factor were also consistent at two time points, that is, the RB group scored higher than the HA group and the HF group. The comparison of the fluid patterns of the three groups led to the conclusion that both the amount and frequency of fluid intake impact this indicator, and the fluid intake pattern of the RB group is the best from this dimension. Similar to the findings, a study conducted among 12 young adults revealed that 1500 mL water supplementation relieved the damage caused by dehydration, including increasing the score of vigor [27]. A systematic analysis concluded that mood was positively influenced by water supplementation [6]. The brain is sensitive to dehydration due to its high water content [37]. Therefore, a decrease in fluid intake may lead to brain dysfunction. The internal mechanism of mood changes may be related to the decrease in heart rate variability and changes in autonomic nervous system function caused by hypohydration [16]. The fluid intake pattern of the RB group was 200 mL/2 h, which satisfies the recommendation of the Dietary Guidelines for Chinese Residents (2016) for adults [28]. This trial proves the rationality of the recommended amount and frequency of daily fluid intake in the dietary guidelines from another point of view. Despite the reasonable fluid intake recommendation and the practical fluid intake tips given in the dietary guidelines, about 20% of the participants in each of three groups were dehydrated at baseline. This indicates that more attention should be paid to increasing the fluid intake of young adults and that health education on recommendations about fluid intake should be intensified.

Among the cognitive performance results, only the portrait memory test scores were found to be statistically significant at time point 1—the higher the score, the better the episodic memory ability of the participants. The HF group had higher scores than the HA group, while the HA group had higher scores than the RB group, which contradicts the results of the POMS test. The RB group performed the worst, and the best group was the HF group. However, there were no statistically significant differences among the three groups at time point 2, this result is similar to a recently published narrative review, which concludes that there is a growing body of evidence that supports the importance of adequate hydration with regard to cognition. However, the evidence lacks consistency, especially relevant to the general healthy population [38]. Another possible explanation is that time may play a role in this process. That is, with the prolongation of time, cognitive performance changes caused by dehydration may naturally alleviate. The internal mechanism that leads to this phenomenon is not clear, but we suggest that it may be related to self-protection and adaptation to the environment. It should be a focus of future research, and the mechanism and degree of the influence of time on cognitive performance and mood should be investigated. Another factor may be the practice effect [39,40]. Although the cognitive tests used in this study were conducted under the principle of randomization when preparing test questions to avoid stimuli repetition and the impact of practice effects as far as possible, it is still possible that each time participants were asked to complete tests they became more familiar with the procedure and a process of learning took place, leading to enhanced performance and higher scores. It is necessary to conduct more studies on the impact of the fluid intake pattern on cognitive performance to draw a precise conclusion.

In terms of the strengths of the study, this is the first time that the effects of fluid intake frequency on cognitive performance and mood have been studied on young adults in China. The study design was a double-blinded, randomized, controlled trial that avoided bias as much as possible; balanced confounding factors; and improved the effectiveness of statistical testing. This type of study is recognized as an effective tool for evaluating intervention measures. This trial was performed with strict quality control, which was achieved by asking the participants to take photos of water against a scale every time they drank, and several investigators were trained and arranged to supervise the participants. The scales used to test cognitive performance and mood are widely accepted by other researchers, and the effectiveness and accuracy of the scales have been verified.

In terms of the weaknesses of the study, the study design did not consider the possible impacts of food source water on participants’ hydration status, mood, and cognitive performance, and there was a lack of baseline data on cognitive performance and mood. The limitations in the design and the lack of relevant data may have put certain restrictions on the interpretation of the influence of the fluid intake pattern on hydration status and mood. At the same time, the intervention period used in this trial was relatively short and does not reflect the long-term effects of different fluid intake behaviors on human health.

## 5. Conclusions

In summary, both the amount and frequency of fluid intake may affect the urine osmolality and vigor (one of the mood indicators), but they only have limited impacts on cognitive performance. Drinking a reasonable amount of water at an appropriate frequency may be beneficial to improve the hydration status and mood of young adults. However, the current results are not robust enough to conclude which fluid intake pattern would be the best to maximize the hydration efficiency and promote human health. Further studies, especially experimental studies, should be designed and conducted from different dimensions and combined with reality to draw causal conclusions and practical suggestions comprehensively.

## Figures and Tables

**Table 1:** The characteristics of the participants.

	Age (years)	Height (cm)	Weight (kg)	Body Mass Index (BMI) (kg/m^2^)
Recommended behavior (RB) group (*n* = 29, 15 males)	19.7 ± 1.3	166.8 ± 7.5	62.4 ± 12.4	21.6 ± 5.6
Half amount (HA) group (*n* = 32, 16 males)	19.7 ± 1.4	165.0 ± 9.0	60.9 ± 10.2	22.4 ± 3.4
High frequency (HF) group (*n* = 31, 15 males)	19.9 ± 1.1	163.1 ± 6.7	60.1 ± 9.9	22.5 ± 3.0
Total (*n* = 92)	19.8 ± 1.2	164.9 ± 7.9	61.0 ± 10.8	22.2 ± 4.1
*p*	0.724	0.190	0.715	0.654

Note: Values are mean ± standard deviation (SD). Differences among the three groups were compared using one-way ANOVA.

**Table 2:** Changes in urine osmolality and hydration status of the three groups.

		UOsm $\left(\mathrm{mOsm}/\mathrm{kg},\;\bar{x\;}\pm \;s\right)$	*p*	Hydration Status (*n* (%))			*p*
				Optimal Hydration	Euhydration	Dehydration	
Baseline	RB group	604 ± 222	0.800	9 (31.0)	14 (48.3)	6 (20.7)	0.564
	HA group	625 ± 238		14 (43.8)	9 (28.1)	9 (28.1)	
	HF group	643 ± 216		10 (32.3)	12 (38.7)	9 (29.0)	
Time Point 1	RB group	646 ± 210 ^a^	<0.001	5 (17.2) ^a^	18 (62.1) ^a^	6 (20.7) ^a^	0.003
	HA group	934 ± 219 ^b^		1 (3.1) ^b^	9 (28.1) ^b^	22 (68.8) ^b^	
	HF group	682 ± 215 ^c^		5 (16.1) ^a,b^	15 (48.4) ^a,b^	11 (35.5) ^a,b^	
Time Point 2	RB group	583 ± 291 ^a^	<0.001	13 (44.8)	7 (24.1)	9 (31.0)	0.058
	HA group	792 ± 281 ^b^		7 (21.9)	10 (31.2)	15 (46.9)	
	HF group	509 ± 244 ^c^		17 (54.8)	10 (32.3)	4 (12.9)	

Note: The difference between the two groups with different superscript letters (^a,b,c^) was statistically significant (*p <* 0.05).

**Table 3:** Comparison of cognitive performance at different timepoints between different groups ($\bar{x}$ ± s).

	Time Point 1			Time Point 2		
	RB Group	HA Group	HF Group	RB Group	HA Group	HF Group
Digit symbol substitution test	66.2 ± 8.9	69.9 ± 9.8	70.2 ± 11.6	75.5 ± 11.3	76.6 ± 9.7	76.4 ± 9.2
Number cancellation test	12.1 ± 2.7	12.1 ± 3.0	11.9 ± 2.8	13.7 ± 2.5	13.3 ± 2.2	13.7 ± 2.4
Working memory span test	9.8 ± 2.0	9.4 ± 1.9	9.6 ± 2.2	10.2 ± 2.0	10.0 ± 1.7	10.1 ± 1.9
Portrait memory test	7.5 ± 5.1 ^#^^†^	13.2 ± 9.1 ^#^*	20.7 ± 9.1 *^†^	22.6 ± 8.4	20.8 ± 7.4	21.8 ± 7.5
Paper-folding test	30.5 ± 6.8	29.2 ± 5.5	29.2 ± 4.8	34.3 ± 5.1	33.4 ± 4.7	35.0 ± 3.3

Note: Values are mean ± standard deviation (SD). ^#^ The difference between the RB group and the HA group was statistically significant; * the difference between the HA group and the HF group was statistically significant; ^†^ the difference between the RB group and the HF group was statistically significant.

**Table 4:** Comparison of mood states at different time points for different groups ($\bar{x}$ ± s).

	Time Point 1			Time Point 2		
	RB Group	HA Group	HF Group	RB Group	HA Group	HF Group
Tension	2.8 ± 2.3	3.2 ± 3.2	3.5 ± 3.7	2.2 ± 2.2	2.8 ± 3.6	3.5 ± 3.7
Anger	2.0 ± 2.7	2.9 ± 4.4	3.1 ± 4.4	1.5 ± 1.9	1.8 ± 3.1	3.1 ± 4.4
Fatigue	2.6 ± 2.8	4.0 ± 4.3	3.9 ± 3.7	2.7 ± 2.7	3.0 ± 3.4	3.9 ± 3.7
Depression	1.7 ± 1.8	2.0 ± 2.7	3.2 ± 4.2	1.1 ± 1.4	1.8 ± 2.8	3.2 ± 4.2
Confusion	2.9 ± 2.8	3.2 ± 2.7	3.7 ± 3.2	2.5 ± 2.5	3.3 ± 3.2	3.7 ± 3.2
Vigor	12.4 ± 4.2 ^#^^†^	9.2 ± 4.9 ^#^	9.0 ± 4.3 ^†^	11.8 ± 4.2 ^#^^†^	9.1 ± 4.7 ^#^	9.0 ± 4.3 ^†^
Esteem	7.4 ± 3.6	6.1 ± 3.7	6.4 ± 3.5	7.5 ± 3.6	5.8 ± 3.4	6.4 ± 3.5
TMD	92.1 ± 13.0	100.0 ± 17.0	101.9 ± 18.8	90.8 ± 12.9	97.8 ± 16.2	101.9 ± 18.8

Note: Values are mean ± standard deviation (SD). ^#^ The difference between the RB group and the HA group was statistically significant; ^†^ the difference between the RB group and the HF group was statistically significant. TMD: total mood disturbance.

## Abbreviations

RB group	Recommended behavior group
HA group	Half amount group
HF group	High frequency group
Uosm	Urine osmolality
CP	Cognitive performance
BMI	Body mass index
TMD	Total mood disturbance
POMS	Profile of Mood State

## Appendix A

**Table A1:** The temperature and humidity during the trial.

	Indoors		Outdoors	
	Temperature (°C)	Humidity (%RH)	Temperature (°C)	Humidity (%RH)
Day 1—10 a.m.	25.1	73.0	23.2	70.0
Day 1—2 p.m.	25.4	72.0	26.2	73.0
Day 1—8 p.m.	24.9	70.0	24.9	72.0
Day 2—10 a.m.	24.4	70.0	25.7	72.0
Day 2—2 p.m.	25.6	71.0	25.8	79.0
Day 2—8 p.m.	25.2	70.0	25.6	73.0
*F*	2.295	2.571	4.550	4.129
*p*	0.204	0.184	0.100	0.112
